# Tripterin liposome relieves severe acute respiratory syndrome as a potent COVID-19 treatment

**DOI:** 10.1038/s41392-022-01283-6

**Published:** 2022-12-24

**Authors:** Haiying Que, Weiqi Hong, Tianxia Lan, Hao Zeng, Li Chen, Dandan Wan, Zhenfei Bi, Wenyan Ren, Min Luo, Jingyun Yang, Cai He, Ailing Zhong, Xiawei Wei

**Affiliations:** Laboratory of Aging Research and Cancer Drug Target, State Key Laboratory of Biotherapy and Cancer Center, National Clinical Research Center for Geriatrics, West China Hospital, Sichuan University, Chengdu, Sichuan People’s Republic of China

**Keywords:** Respiratory tract diseases, Infectious diseases

## Abstract

For coronavirus disease 2019 (COVID-19), caused by severe acute respiratory syndrome coronavirus 2 (SARS-CoV-2), 15–30% of patients are likely to develop COVID-19-related acute respiratory distress syndrome (ARDS). There are still few effective and well-understood therapies available. Novel variants and short-lasting immunity are posing challenges to vaccine efficacy, so finding antiviral and antiinflammatory treatments remains crucial. Here, tripterin (TP), a traditional Chinese medicine, was encapsulated into liposome (TP lipo) to investigate its antiviral and antiinflammatory effects in severe COVID-19. By using two severe COVID-19 models in human ACE2-transgenic (hACE2) mice, an analysis of TP lipo’s effects on pulmonary immune responses was conducted. Pulmonary pathological alterations and viral burden were reduced by TP lipo treatment. TP lipo inhibits SARS-CoV-2 replication and hyperinflammation in infected cells and mice, two crucial events in severe COVID-19 pathophysiology, it is a promising drug candidate to treat SARS-CoV-2-induced ARDS.

## Introduction

SARS-CoV-2 has resulted in a formidable outbreak worldwide. According to the WHO coronavirus (COVID-19) dashboard, the number of confirmed cases has already reached 605 million by September 2022. Moreover, a cumulative total of 6.4 million deaths have been reported to the WHO. Of note, the most severe form of COVID-19 results in life-threatening acute lung injury (ALI) and acute respiratory distress syndrome (ARDS) that is mostly accompanied by cytokine storm and has a high mortality rate of 40–50%.^1,2^ The COVID-19 patients with ARDS showed hyperinflammatory response, lymphopenia, microthrombosis and diffuse alveolar damage and extensive pulmonary injury.^3,4^ Although vaccination remains one of the most efficient ways to contain the pandemic, the limited vaccine effectiveness against SARS-CoV-2 variants makes it a long-lasting challenge to slow the spread of the virus. Therefore, in the present stage of the battle against COVID-19, a major purpose is to develop antiviral drugs that could reduce the severity and mortality of the disease. As a result, considerable efforts have been devoted to developing drugs targeting the SARS-CoV-2/COVID-19 machinery.^5^ For example, remdesivir has been approved by the FDA for the treatment of mild-to-moderate COVID-19. However, to date, none of the clinically assessed antiviral drugs have manifested significant efficacy against COVID-19.^6^ In addition, according to the COVID-19 treatment guidelines from the National Institutes of Health (NIH), the antiinflammatory corticosteroid dexamethasone is the NIH-recommended therapeutic method for the management of severe symptoms associated with SARS-CoV-2 infection. In a controlled, open-label trial, it was shown that the use of dexamethasone results in lower 28-day mortality in COVID-19 patients with ARDS. However, this treatment method is coupled with opportunistic nosocomial infections and immunosuppression.^7^ Hence, it is plausible that novel therapies that combine the advantages of antiviral drugs and anti-inflammatory corticosteroids would be the ideal treatment method against severe COVID-19.

The constant emergence of new variants of SARS-CoV-2 and the large expenditures on the development of COVID-19-specific drugs have driven researchers to seek the possibility of modifying or repurposing licensed drugs capable of alleviating inflammation and inhibiting viral replication from fighting against COVID-19. In particular, natural products have been widely used to treat respiratory infectious diseases and inflammatory diseases.^8,9^ However, jeopardized by the relatively poor kinetic properties, such as the weak absorption ability and large molecular weight, natural products are often associated with low bioavailability and unsatisfactory safety profiles. Nonetheless, boosted by the recent progress that has been made in terms of nanotechnology, the conjugation of natural products and nanovectors has been shown to manifest increased delivery efficiency and ameliorated toxicity, thereby providing a new therapeutic strategy for the treatment of an array of diseases.^10^ However, few studies have been conducted to evaluate the antiinflammatory effect and the antiviral activity of natural products against SARS-CoV-2 infection in animal models.

Tripterin (TP) is a natural product isolated from the herb *Tripterygium wilfordii*. Previous studies have demonstrated that TP exerts antiinflammatory and antiinfective effects.^11,12^ However, TP has several drawbacks, such as low permeability, poor solubility, off-target side effects, and limited oral bioavailability.^13–16^ Since TP is highly hydrophobic, the toxic dimethyl sulfoxide (DMSO) is commonly used to dissolve TP in preclinical studies.^17,18^ TP is yet to be commonly used in clinical settings. In addition, the mechanism of the antiinflammatory effects of TP is still unclear. Liposomes have a bilayer structure that enables the loading of hydrophobic TP for better solubility and bioavailability and lower toxicity.^19^ Liposomes coated with polyethylene glycol (PEG) have the ability to improve systemic circulation time and decrease immunogenicity.^19,20^ Wolfram et al. have reported that TP embedded in a liposomal bilayer are released over a prolonged period of time, which increases the bioavailability of the agent and reduces the dosing frequency.^19^ Our preliminary studies found that Tripterin liposome (TP lipo) could inhibit LPS-induced pulmonary inflammation. More importantly, a previous in vitro study reported that TP could bind to the main protease (M^pro^) and receptor-binding domain (RBD) of SARS-CoV-2 and is able to reverse gene expression signature and inhibit the virus replication in SARS-CoV-2-infected cells.^21^ Echoing these findings, we managed to assess the therapeutic value of TP lipo for the treatment of COVID-19.

In the current study, the antiinflammation effects exerted by TP lipo were evaluated in inactivated and live SARS-CoV-2-induced ARDS models. It is worth noting that TP lipo remarkably alleviated pulmonary inflammation and efficiently inhibited the viral replication rate and infectivity in vivo and in vitro. These results indicate that TP lipo is a dual-effect drug candidate for the treatment of COVID-19. Moreover, based on single-cell RNA sequencing (scRNA-seq), we further analyzed the alterations of the immune microenvironment in TP lipo-treated mice as well as the mechanisms by which TP lipo induces pulmonary immunoregulation. In summary, our work suggests that TP lipo holds promise for further expanding the toolbox of treatment methods against severe COVID-19.

## Results

### In vitro and in vivo characterizations of TP lipo

**Fig. 1:**
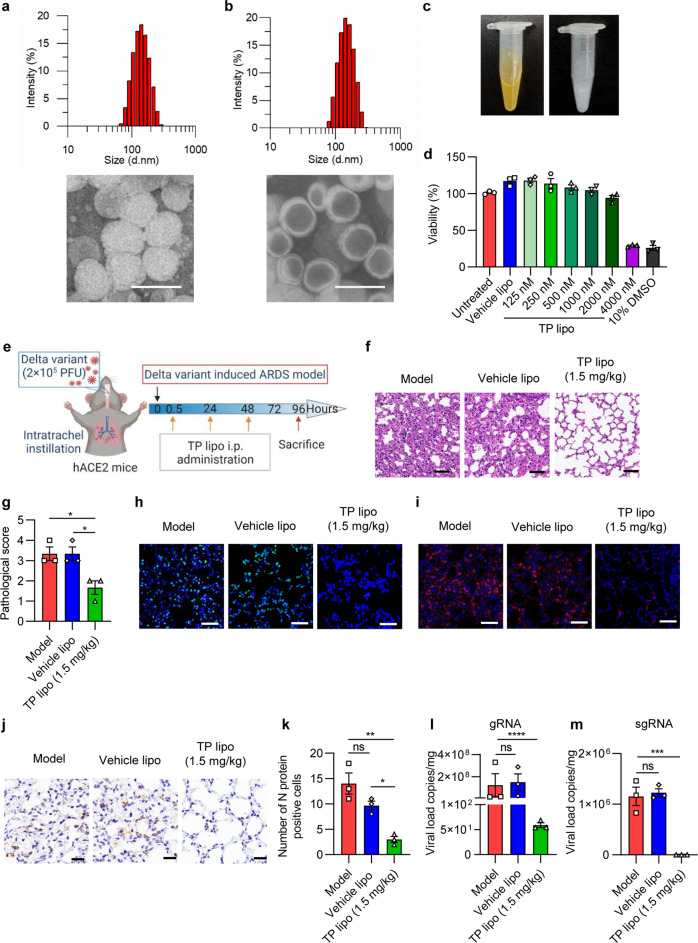
In vitro and in vivo characterizations of TP lipo. **a**, **b** Hydrodynamic diameter of TP lipo (**a**) and Vehicle lipo (**b**), measured from DLS. TEM images of TP lipo (**a**) and Vehicle lipo (**b**). The scale bar represents 200 nm. **c** Photographs of TP lipo (left) and Vehicle lipo (right). **d** Cell viability of Vero E6 cells influenced by Vehicle lipo, TP lipo at different concentrations, and 10% DMSO. **e** Protocol of the hACE2 mouse model of ARDS induced by the Delta variant. H&E staining (**f**) and pathological score (**g**) in representative mouse lung sections. The scale bar represents 50 μm. **h** Images of TUNEL labeling for cell apoptosis analysis. The scale bar represents 50 μm. **i** Typical images of immunofluorescence analysis for Ly6G-positive neutrophils. The scale bar represents 50 μm. **j** Immunohistochemical staining of nucleocapsid (N) protein in the lungs of Delta variant-infected hACE2 mice. **k** Histogram of the number of N proteins in representative mouse lung sections. qRT-PCR quantification of Delta variant gRNA (**l**) and sgRNA (**m**) in infected mice with or without TP lipo treatment. Data represent the mean ± SEM; *n* = 3. Significance is indicated by: ns no significance; *P ≤ 0.05; **P ≤ 0.01; ***P ≤ 0.005; ****P ≤ 0.0001

The TP lipo was produced by thin film hydration and was extruded through different porn-size polycarbonate membranes. The DLS indicated that the hydrodynamic diameter was ~134.3 nm for TP lipo (Fig. 1a) and ~142.0 nm for the vehicle (Fig. 1b). In addition, the formulation of TP lipo presents as a yellow, translucent solution, while the color of Vehicle liposomes (Vehicle lipo) without TP is white (Fig. 1c). Furthermore, TEM was employed to determine the appearances and shapes of TP lipo and Vehicle lipo. The TEM images illustrated that Vehicle lipo is hollow spherical particles (Fig. 1b), but TP lipo are filled with nanosized substances, indicating that TP is coated by liposomes (Fig. 1a). In addition, based on the results of high-performance liquid chromatography (HPLC), TP lipo had an entrapment efficiency (EE%) of 74.45 ± 5.36%.

To assess the cytotoxicity of TP lipo, for 48 h following treatment with Vehicle lipo or different concentrations of TP lipo, Vero E6 cells were assessed for viability using the CCK-8 assay. It was observed that Vehicle lipo is nontoxic to Vero E6 cells. Additionally, the cytotoxicity of TP lipo in a concentration range from 125 to 2000 nM is also negligible. However, when the concentration surpassed 4000 nM, TP lipo significantly unpaired the cell viability (Fig. 1d). Through intratracheal instillation of the Delta variant SARS-CoV-2 virus (2 × 10^5^ PFU) in human ACE2-transgenic mice (hACE2), we developed ARDS models that mimic severe COVID-19.^22^ Following that, we investigate TP lipo’s efficacy against ARDS induced by SARS-CoV-2 Delta variant infection in vivo. Mice were injected with Vehicle lipo or TP lipo (1.5 mg/kg) after instilling the virus intratracheally 0.5, 24, and 48 h later (Fig. 1e). The mice were sacrificed 96 h after being exposed to the Delta variant to assess the effects of TP lipo on ARDS. Haematoxylin and eosin (H&E)-stained lung sections showed that the administration of TP lipo at 1.5 mg/kg significantly ameliorated pulmonary inflammation (Fig. 1f, g). Additionally, the terminal deoxynucleotidyl transferase-mediated dUTP nick-end labeling (TUNEL) assay showed that TP lipo reduced the apoptosis level in virus-challenged mice (Fig. 1h). By measuring the level of Ly6G, we showed that TP lipo inhibited the accumulation of neutrophils in the lung (Fig. 1i). It is essential for virion assembly and viral replication that the nucleocapsid (N) protein encapsulates the SARS-CoV-2 genome.^23–25^ The level of N protein in TP lipo-treated mice was found to be lower than that in untreated mice based on the results of immunohistochemistry (IHC) (Fig. 1j, k). The lung tissues were also collected for quantitative real-time polymerase chain reaction (qRT-PCR) 96 h after infection. The mean copy numbers of genomic RNA (gRNA) in the Model group and Vehicle lipo group were 1.24 × 10^8^ and 1.51 × 10^8^ copies/mg lung tissue, respectively (Fig. 1l). The mean copy numbers of viral subgenomic RNA (sgRNA) in the Model group and the Vehicle lipo group were 1.16 × 10^6^ and 1.23 × 10^6^ copies/mg lung tissue, respectively (Fig. 1m). In contrast, both pulmonary Delta variant gRNA and sgRNA levels were undetectable in the TP lipo-treated group, which indicated that TP lipo could significantly interfere with viral replication in vivo.

### The antiviral effects of TP lipo on Vero E6 cells infected with the Delta variant

**Fig. 2:**
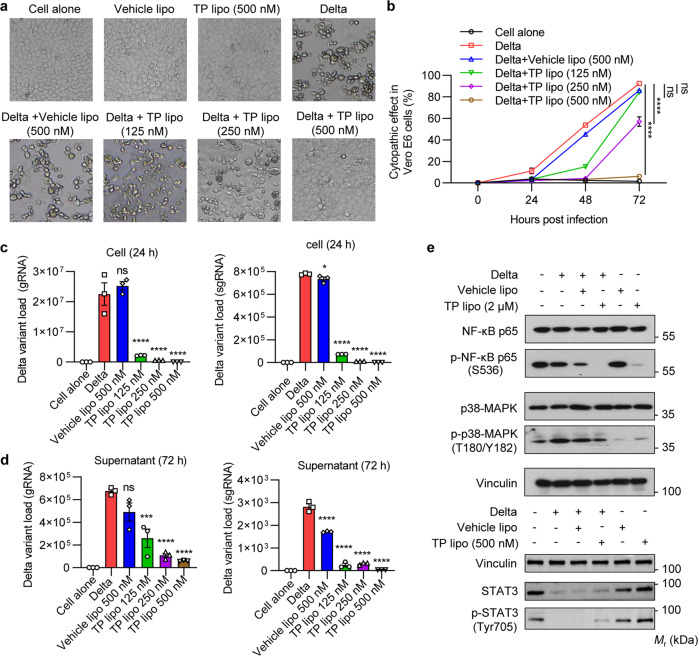
TP lipo inhibits Delta variant in vitro propagation in Vero E6 cells. Vero E6 cells (5 × 10^4^) were preincubated with TP lipo for 1 h at 37 °C and then exposed to the Delta variant (MOI = 0.05). **a** Representative CPE pictures estimated at 72 h postinfection are shown under a microscope. **b** The percentage of CPE at 24, 48, and 72 h was analyzed from three independent experiments. **c** qRT-PCR quantification of Delta variant gRNA and sgRNA in infected Vero E6 cells at 24 h postinfection. **d** qRT-PCR quantification of Delta variant gRNA and sgRNA in supernatants at 72 h postinfection. **e** Delta variant-infected Vero E6 cells were exposed to Vehicle lipo or a different dose of TP lipo for up to 24 h (MOI = 0.05). Total protein was extracted and subjected to analysis of three phosphorylated protein levels, including p-NF κB p65, p-p38 MAPK, and p-STAT3. Vinculin was used as a loading control. Data represent the mean ± SEM; *n* = 3. Significance is indicated by: ns no significance; *P ≤ 0.05; ***P ≤ 0.001; ****P ≤ 0.0001

In order to determine whether TP lipo has antiviral effects in vitro, a total of 5 × 10^4^ Vero E6 cells were preincubated with Vehicle lipo or different concentrations of TP lipo for 1 h at 37 °C, followed by exposure to the Delta variant (MOI = 0.05). After 72 h, the cytopathic effects (CPE) were evaluated under a microscope (Fig. 2a). When exposed to the virus for 72 h, the results showed the percentage of CPE in 500 nM TP lipo-treated cells (6.0%) was much lower than that in Vehicle lipo-treated (86.1%) or untreated cells (92.4%). Moreover, the CPE of infected cells was suppressed by TP lipo in both time- and dose-dependent manners (Fig. 2b). In addition, to assess the influence of TP lipo on viral replication, we measured the transcript levels of Delta variant sgRNA and gRNA in Vero E6 cells at 24 and 72 h after TP lipo infection using a qRT-PCR assay. It was found that 125, 250, or 500 nM TP lipo efficiently inhibited the RNA levels of gRNA and sgRNA at both time points, suggesting that TP lipo could effectively repress the viral loads and replication of the Delta variant (Fig. 2c, d). In addition, the western blotting (WB) results further demonstrated that TP lipo inhibited the activation of inflammation-associated proteins (NF-κB, p38 MAPK) and the dephosphorylation of protein p-STAT3 in Delta variant-infected Vero E6 cells^26–28^ (Fig. 2e).

### TP lipo ameliorates ARDS induced by FA-S in hACE2 mice

**Fig. 3:**
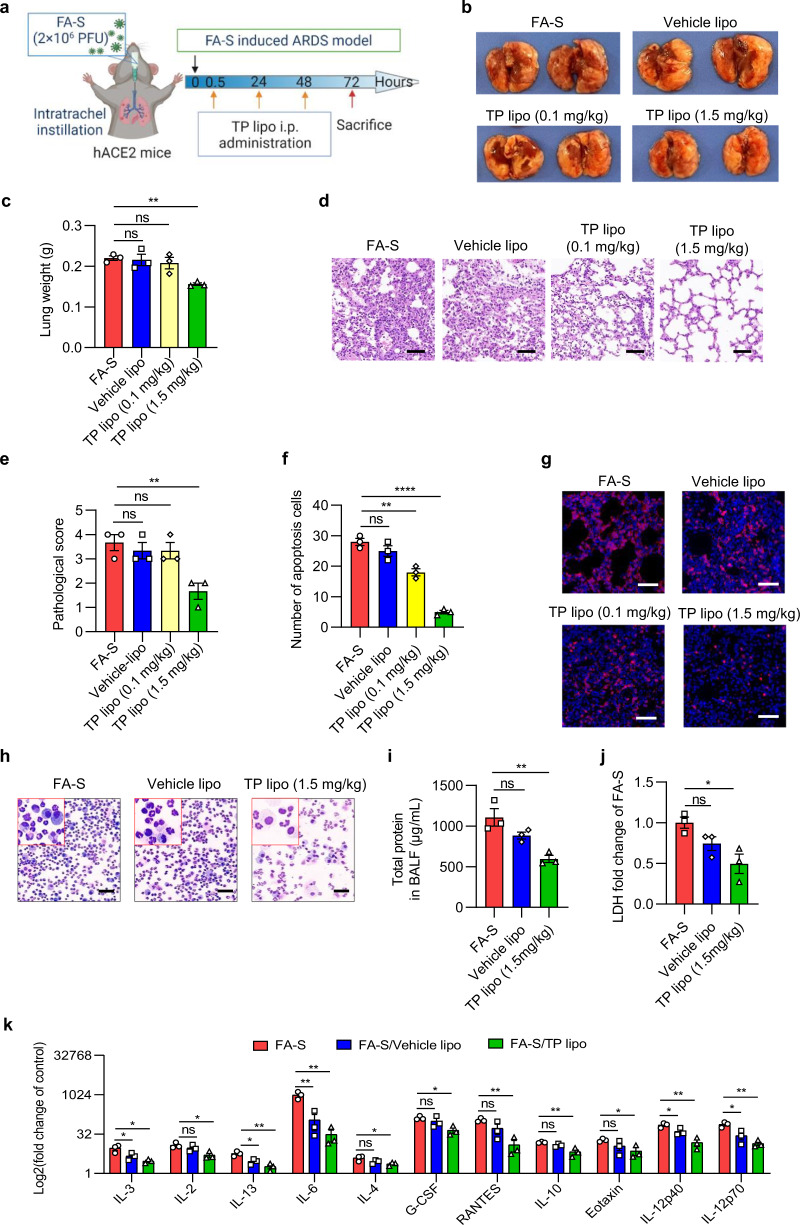
TP lipo ameliorates ARDS induced by FA-S in hACE2-KI mice on a C57BL/6 J background. **a** Protocol of the hACE2 mouse ARDS model induced by FA-S. **b**, **c** Images of lung tissues (**b**) and changes in lung weight (**c**). **d**, **e** H&E staining (**d**) and pathological score (**e**) in representative mouse lung sections. The scale bar represents 50 μm. **f** Number of apoptotic cells obtained from TUNEL staining. **g** Typical images of immunofluorescence analysis for Ly6G-positive neutrophils. The scale bar represents 50 μm. **h** Typical images of cells in BALF obtained from Diff-Quik staining. The scale bar represents 50 μm. **i**, **j** Total protein level (**i**) and fold change of LDH (**j**) in BALF. **k** Comparison of key proinflammatory cytokine responses in FA-S infection compared with Vehicle lipo and TP lipo. The results are presented as log2 of the fold change of the control. Data represent the mean ± SEM; *n* = 3. Significance is indicated by: ns no significance; *P ≤ 0.05; **P ≤ 0.01; ****P ≤ 0.0001

To verify the ameliorative effects of TP lipo on ARDS induced by the infection of wild-type (WT) SARS-CoV-2, ARDS models were established by intratracheal instillation of formaldehyde-inactivated wild-type SARS-CoV-2 (FA-S) (2 × 10^6^ PFU) in hACE2 mice according to our previous work.^29^ The inactivated WT SARS-CoV-2 has no infection ability in hACE2 mice but is an effective stimulus for an intense inflammatory response.^29^ Then, the Vehicle lipo or different dosages of TP lipo were intraperitoneally administered to mice 0.5, 24, and 48 h after the intratracheal instillation of the virus (Fig. 3a). Then, the mice were sacrificed 72 h after intratracheal instillation, and the lungs were isolated for the evaluation of pathological characteristics. We found that only the lungs treated with 1.5 mg/kg TP lipo, but not those treated with 0.1 mg/kg TP lipo, showed smaller size, fewer patches of hemorrhage, and lower levels of bilateral congestion and edema after viral infection. In contrast, the appearance of lungs from Vehicle lipo-treated mice largely recapitulates those from untreated mice after viral infection (Fig. 3b). Additionally, as shown in Fig. 3c, 1.5 mg/kg TP lipo also reduced lung weight in FA-S-induced ARDS mice. Moreover, the H&E staining of lung sections showed that the administration of TP lipo at 1.5 mg/kg significantly ameliorated the pulmonary inflammation, which is illustrated by the decreased inflammatory cell infiltration, lower interstitial edema, necrotic debris, and pathological score (Fig. 3d, e). Additionally, the TUNEL assay showed that TP lipo reduced the number of apoptotic cells in virus-challenged mice (Fig. 3f). In addition, the neutrophils in lung sections were stained positive for Ly6G. As a hallmark of ARDS, neutrophil infiltration was repressed significantly by 1.5 mg/kg TP lipo (Fig. 3g).

To assess neutrophil infiltration into alveoli, bronchoalveolar lavage fluid (BALF) was also collected from mice. In FA-S-induced ARDS mouse models, diff-Quik staining of cells in BALF showed that 1.5 mg/kg TP lipo suppresses neutrophil infiltration into alveoli at 3 days post-instillation (dpi) (Fig. 3h). Furthermore, BALF levels of total protein and lactate dehydrogenase (LDH) were also lower in TP lipo-treated mice than in untreated or Vehicle lipo-treated mice (Fig. 3i, j). Moreover, proinflammatory cytokines are elevated in BALF, including IL-3, IL-2, IL-13, IL-6, IL-4, G-CSF, RANTES, IL-10, Eotaxin, IL-12p40, and IL-12p70, also represent ARDS. Here, we showed that the levels of these cytokines were reduced by TP lipo treatment (Fig. 3k). Together, these findings suggest that TP lipo could potently ameliorate ARDS induced by FA-S in hACE2 mice.

### TP lipo ameliorates ARDS induced by LPS in WT mice

To further validate the ameliorative effect of TP lipo on ARDS, a different ARDS model was established by postlingually instilling 5 mg/kg lipopolysaccharide (LPS) in C57BL/6 J WT mice. Then, mice, after instilling LPS, were injected with Vehicle lipo or different dosages of TP lipo intraperitoneally 0.5, 24, and 48 h later (Supplementary Fig. S1a). We isolated the lungs from all mice 72 h after instillation and evaluated their pathological characteristics. Again, it was found that the lungs treated with 1.5 mg/kg TP lipo showed smaller size, fewer patches of hemorrhage, lower levels of bilateral congestion, edema, and lower weight (Supplementary Fig. S1b, c). In addition, according to lung images of H&E staining, TP lipo-treated mice had decreased inflammatory cell infiltration, less interstitial edema, and necrotic debris (Supplementary Fig. S1d) with an average pathological score of 0-1 in their lungs (Supplementary Fig. S1e). Additionally, TP lipo reduced the apoptosis level in LPS-treated mice (Supplementary Fig. S1f). Neutrophil infiltration was repressed by 1.5 mg/kg TP lipo (Supplementary Fig. S1g). In addition, the Diff-Quick staining of cells in BALF at 3 dpi showed that 1.5 mg/kg TP lipo suppresses the infiltration of neutrophils into alveoli in LPS-induced ARDS mouse models (Supplementary Fig. S1h). Furthermore, in TP lipo-treated mice, total protein and LDH release in BALF were significantly lower than in untreated or Vehicle lipo-treated mice (Supplementary Fig. S1i, j). These results revealed that TP lipo has a strong antiinflammatory ability in viruses and bacteriotoxin-induced ARDS.

### Pulmonary cell types in hACE2 mouse ARDS revealed by scRNA-seq

**Fig. 4:**
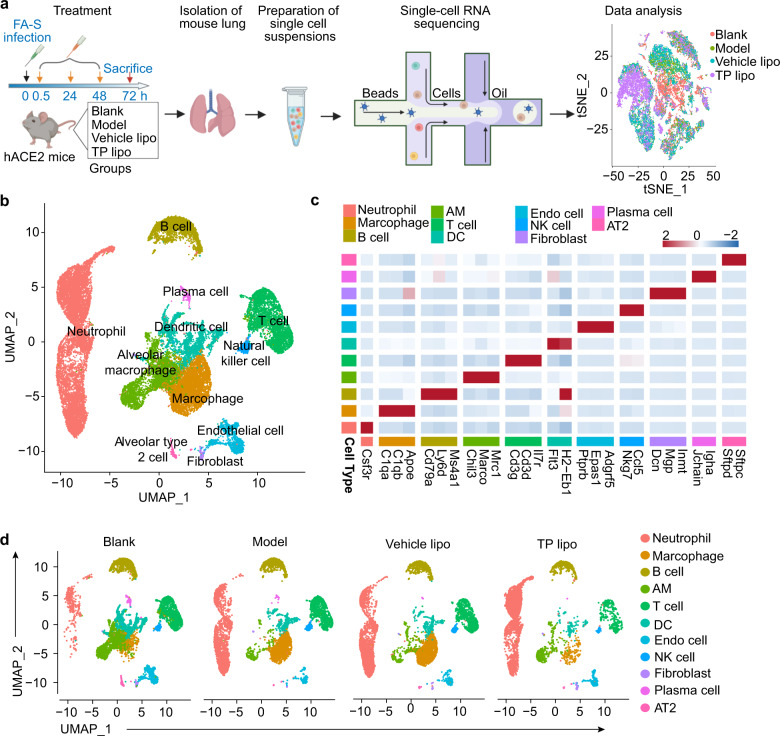
scRNA-seq analysis of pulmonary cell clusters in treated mice. **a** Schematic illustration of the treatment strategy, lung dissociation, single-cell preparation, and scRNA-seq sequencing and analysis. **b** UMAP plot of 28229 cells from Blank, Model, Vehicle lipo, and TP lipo mouse testes. The number of cells in each cluster: Neutrophil (9308), Macrophage (3932), B cell (2977), AM (3592), T cell (3499), DC (2676), Endo cell (1226), NK cell (429), Fibroblast (220), Plasma cell (193), and AT2 (177). **c** Heatmap of the top transcripts in each cluster showed clear demarcation between different clusters. **d** UMAP plots showing the dynamic changes in all cell clusters in the lung among each group

Pulmonary single cells from the four groups were collected 72 h after FA-S infection. The immune response to FA-S infection and TP lipo treatment was assessed by scRNA-seq (Fig. 4a). Using the graph-based method and uniform manifold approximation and projection (UMAP), 28,229 cells from four groups were plotted, and 11 cell clusters were identified (Fig. 4b). Based on Supplementary Fig. S2a, the four experimental groups showed equivalent cellular distributions and no enriched populations for batch variation or technical variation. Generally, the top three cell types in mouse lungs were neutrophil (9308), macrophage (3932), and alveolar macrophage (AM) (3592) (Fig. 4b). Genes associated with particular cell types were used to annotate clusters of cells, as indicated by the gene expression heatmap (Fig. 4c). Mean expression patterns of key signature genes, such as *Csf3r* (Neutrophil), *C1qa* (Macrophage), *Cd79a* (B cell), *Flt3* (Dendritic cell, DC), *Macro* (AM), and *Cd3g* (T cell), were also projected on the UMAP plot (Supplementary Fig. S2b). Compared with the Model and the Vehicle lipo groups, it was clearly evident that TP lipo reduced neutrophil and macrophage recruitment to the lungs and increased AM cell residence in them (Fig. 4d). Thus, TP lipo may alleviate pulmonary inflammation by changing the lung immune microenvironment.

### TP lipo treatment downregulates gene signatures of macrophage inflammation in FA-S-infected hACE2 mice

**Fig. 5:**
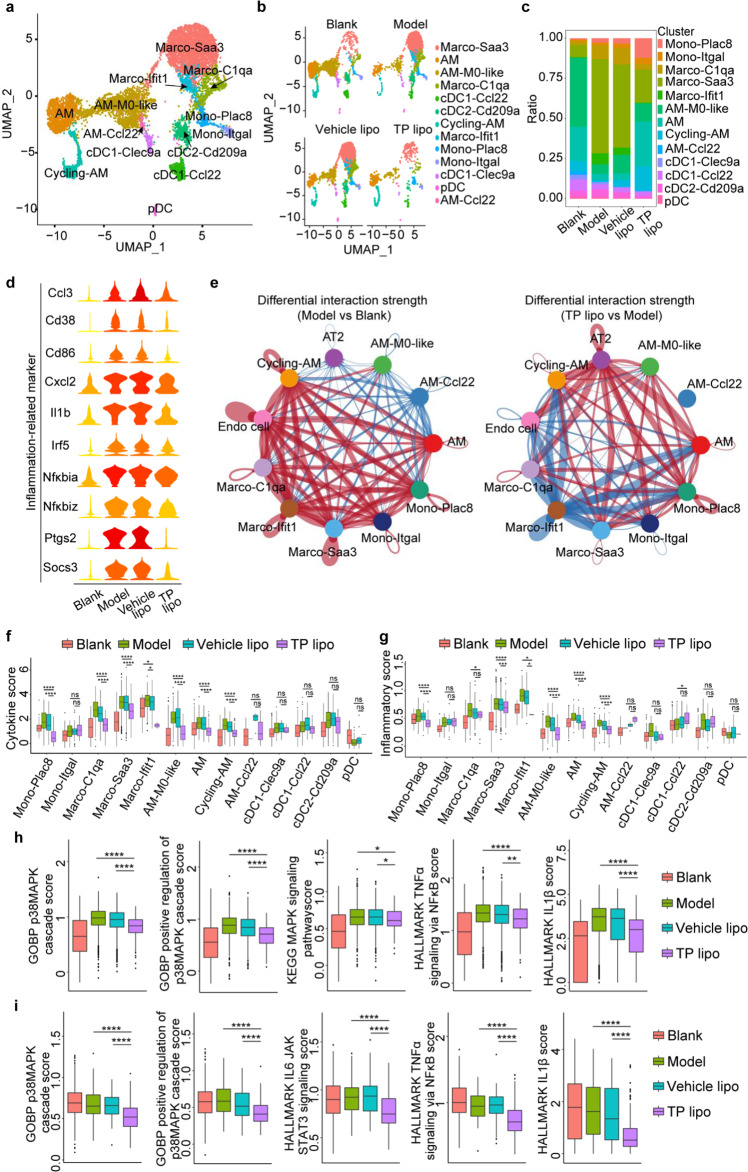
TP lipo alleviates severe pulmonary inflammation by reducing pulmonary recruitment of the Macro-Saa3 cell subset and increasing pulmonary residency of the AM cell subset**. a** UMAP plot of 13 subsets of myeloid cells colored by cluster identity. **b** UMAP plots showing the dynamic changes in the myeloid cell clusters among each group. **c** Ratio of all cells belonging to the myeloid cell clusters. **d** Violin plots showing the smoothed expression distribution for selected inflammation-related marker genes for each group. Red, high expression; yellow, low expression. **e** Differential interaction strength of significant ligand-receptor pairs between any pair of two cell populations. The edge width was proportional to the indicated strength of ligand-receptor pairs. Blue edge, weakened cellular communication; Red edge, strengthened cellular communication. **f**, **g** The cytokine score (**f**) and inflammatory score (**g**) of myeloid cell subsets from Blank controls, Model, Vehicle lipo, and TP lipo groups. **h**, **i** Five inflammatory-related pathway scores of Macro-Saa3 (**h**) and AM (**i**) among Blank controls, Model, Vehicle lipo, and TP lipo groups. Significance is indicated by: ns no significance; **P ≤ 0.01; ***P ≤ 0.005; ****P ≤ 0.0001

To investigate the effects of TP lipo on macrophage inflammation, we divided 8436 pulmonary myeloid cells into 13 subsets by Harmony integration and cluster analysis (Fig. 5a), representing macrophages, AMs, DCs, and monocytes (Supplementary Data S1 and Fig. 5b). According to the highly expressed inflammation-related gene *Saa3*, complement-related gene *C1qa*, and interferon stimulation-related gene *Ifit1*, macrophages were divided into 3 distinct subclusters (Macro-Saa3, Macro-C1qa, and Macro-Ifit1) (Supplementary Fig. S3a, b). At 3 dpi, compared with the ARDS model, the Macro-Saa3 subset and the AM subset in TP lipo-treated mice changed dramatically (Fig. 5b). During acute inflammation, serum amyloid A3 (SAA3) plays a role as an acute phase response protein. IL-6 and TNF-α are upregulated by SAA3 by activating the p38 and NF-κB pathways in a MyD88-dependent manner.^30^ The ratio of cluster Macro-Saa3 in TP lipo-treated mice (20.6%) was much lower than that in untreated mice (58.7%), indicating that TP lipo could alleviate inflammation by reducing the recruitment of Macro-Sas3 cells to the lung (Fig. 5c). Based on the differentially expressed genes *Macro* (a specific marker of alveolar macrophages)^31^ and *Mrc1* (encoding CD206, a marker of type II macrophages)^32^ (Fig. 5c), we speculated that AM is a group of M2-like AM cell subset with an anti-inflammatory phenotype. Reportedly, there is a higher endosomal pH in M2-like AMs, but lower lysosomal pH, which may limit virus diffusion by destroying it in the lysosomes.^33^ The ratio of the AM subset in TP lipo-treated mice (27.9%) was significantly higher than that in ARDS Model mice (3.8%) (Fig. 5c). Thus, it could be inferred that TP lipo limits SARS-CoV-2 spread and ameliorates ARDS by maintaining the number and function of AMs. Furthermore, the cell-cell communication strength of endothelial (Endo) cells and type II alveolar epithelial (AT2) cells to other cell clusters were analyzed by CellChat (Fig. 5e). After FA-S infection, macrophages, AM, and monocyte showed strong communication with Endo cell, which could be ascribed to the inflammatory endothelial cell phenotype contributed by the cytokine storm.^34^ However, those strong communications could be weakened by TP lipo (Fig. 5e). TP lipo treatment increased the communication strength between AT2 cells and AM cell subset, suggesting that TP lipo may sustain the resident alveolar stem cell properties of AT2 cells to induce the proliferation and repair of lung alveolar type I epithelial cells (AT1) after injury.^35^

To explore the antiinflammatory effect of TP lipo on myeloid cells, we assessed the expression distribution of several inflammation-related genes, such as *Ccl3*, *Cd38*, *Cd86*, *Cxcl2*, *Il1b*, *Irf5*, *Nfκbia*, *Nfκbiz*, *Ptgs*, and *Socs3*, with or without TP lipo treatment. Gene expression levels were clearly decreased after TP lipo treatment (Fig. 5d). To further understand the levels of cytokine storms at each cell subset level, we defined a cytokine score and inflammatory score based on well-defined genes to assess the cytokine score and inflammation score in each cell. Most cell subsets had elevated scores after FA-S infection, especially three subclusters of macrophages (Macro-C1qa, Macro-Saa3, and Macro-Ifit1), indicating that these cells are the main source of the inflammatory cytokine storm. TP lipo significantly blocked this inflammatory response (Fig. 5f, g). Five inflammatory-related pathway scores were calculated on clusters of Macro-Saa3 and AM, to determine the pathways affected by TP lipo administration. The results showed that TP could ameliorate pulmonary inflammation by interfering with the GOBP p38 MAPK cascade reaction, GOBP positive regulation of the p38 MAPK cascade pathway, IL-6 JAK STAT3 signaling, TNFα signaling via NFκB, and IL-1β signaling in Macro-Saa3 (Fig. 5h) and AM subsets (Fig. 5i). Other proinflammatory functional pathways, such as the IFNα response, IFNγ response, and complement, were also downregulated by TP lipo based on gene set enrichment analysis (GSEA). Remarkably, IL-2 and IL-6 are important immunological parameters associated with long COVID-19.^36,37^ Collectively, TP lipo reduced the recruitment of inflammatory macrophages, especially Macro-Saa3 cells, and enhanced the residence of M2-like AM cell subset in the lung, contributing to the remission of lung inflammation.

### Neutrophil-Ccl3 cells play a dominant proinflammatory role in FA-S-induced ARDS

**Fig. 6:**
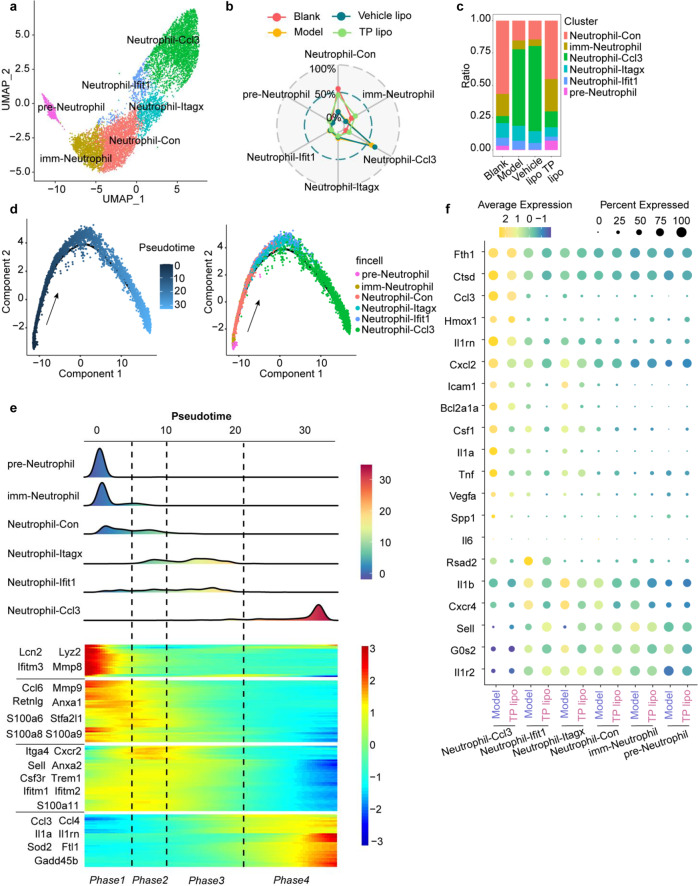
Neutrophil-Ccl3 is the main inflammatory neutrophil in the mouse lung of the FA-S-induced ARDS model. **a** UMAP plot of six subsets of neutrophils colored by cluster identity. **b** Radar chart indicating the relative expression of each neutrophil cell population. **c** Ratio of all cells belonging to the neutrophil clusters. **d** Pseudotime trajectory analysis of the neutrophil clusters. **e** Heatmap of the gene expression dynamics during neutrophil differentiation, following the trajectory timeline shown in (**d**). Left, representative enriched genes; right, the gene group. **f** Dot plot showing the scale expression of selected signature genes for two groups based on six subsets of neutrophils. Yellow, high expression; Green, low expression; Size, the proportion of neutrophil subpopulations expressing transcription factors

A total of 9024 pulmonary neutrophils were reclustered and separated into 6 subpopulations on the UMAP map according to the classical marker genes reported previously (Supplementary Data S1 and Figs. S4a, 6a).^38^ The cluster pre-Neutrophils highly expressed marker genes of bone marrow neutrophils (*Cd177*)^39^ and secondary granule proteins (*Ltf*, *Cybb*).^38,40^ Thus, cluster pre-Neutrophil could be more primitive neutrophils (Supplementary Fig. S4a, 6d). There was an enrichment of tertiary (gelatinase) granule markers (*Mmp8*, *Mmp9*) in the imm-Neutrophil cluster, indicating these neutrophils may be immature neutrophils since they are shifting from the metamyelocyte stage to the band stage^41^ (Supplementary Fig. S4a). We also observed the enrichment of genes encoding antimicrobial peptides and neutrophil mobilization (*S100a8*, *Csf3r*, *Cxcr2*), suggesting that cluster Neutrophil-Con may be conventional neutrophils (Supplementary Fig. S4a). While cluster Neutrophil-Con and cluster imm-Neutrophil were mainly present in both normal and TP lipo treatment conditions, cluster Neutrophil-Ccl3 was specific to the FA-S-induced ARDS condition and thus important in accelerating inflammatory development (Fig. 6b, c). We used Monocle to place differentiating neutrophil populations in pseudotime. There was a tightly organized trajectory during neutrophil differentiation and maturation, starting from pre-Neutrophil cells and ending with Neutrophil-Ccl3 cells (Fig. 6d). Neutrophils with high expression of *Itgax* (Neutrophil-Itagx) and *Ifit1* (Neutrophil-Ifit1) are in an evolutionary transition period (Fig. 6d).

To understand the molecular events involved in neutrophil evolution, we identified four distinct gene expression dynamics following the trajectory timeline (Fig. 6e). Phase 1 genes comprised classic neutrophil markers with specific granules (*Lcn2*, *Lyz2, Mmp8*, and *Mmp9*), neutrophil maturation (*Retnlg*, *Ccl6*, *Stfa2l1*, and *S100a6*) and chemotaxis (*S100a8* and *S100a9*).^40,42^ Due to the high expression of neutrophil maturation and aging genes (*Anxa2*, *Sell*, *Ifitm1*, and *S100a11*) and chemotaxis genes (*Cxcr2*, *Csf3r*, and *Trem1*), phase 2 and 3 neutrophils were more mature and chemotactic than phase 1 cells, in line with their transition state before inflammation. Many proinflammatory genes were enriched in phase 4 concerning the inflammatory response (*Ccl3*, *Ccl4*, *Il1α*, *Il1*, and *Il1rn*) and reactive oxygen species (ROS) (*Sod2*), suggesting that Neutrophil-Ccl3 cells possess robust anti-infective capabilities. Notably, *Gadd45b*, encoding the stress sensor protein Gadd45b, may strengthen the innate immune function of Neutrophil-Ccl3 by maintaining inflammatory stimuli-mediated chemotaxis.^43^

We next determined whether TP lipo treatment affected the gene expression of neutrophil-derived proinflammatory cytokines and chemokines in severe COVID-19 cases.^44,45^ TP lipo treatment significantly inhibited the expression of the genes *Ccl3*, *Il1rn*, *Cxcl2*, *Csf1*, *Il1a*, *Tnf*, *Il6*, *Il1r2*, *Icam1*, and *Cxcr4* in Neutrophil-Ccl3 cells (Fig. 6f), indicating that TP lipo played an important role in blocking the cytokine storm. Additionally, TP lipo could reduce neutrophil infiltration by downregulating the gene expression of *Icam1* (neutrophil adhesion) and *Vegfa* (angiogenesis).^46,47^ TP lipo might relieve endotheliopathy and inflammation in long COVID-19 patients by targeting IL-6 and ICAM-1.^48^ Collectedly, Neutrophil-Ccl3 cells are essential in responding to FA-S-induced ARDS, and TP lipo can target them well and effectively alleviate hyperinflammation.

### TP lipo treatment abolishes inflammatory cytokines in neutrophils of FA-S-infected hACE2 mice

**Fig. 7:**
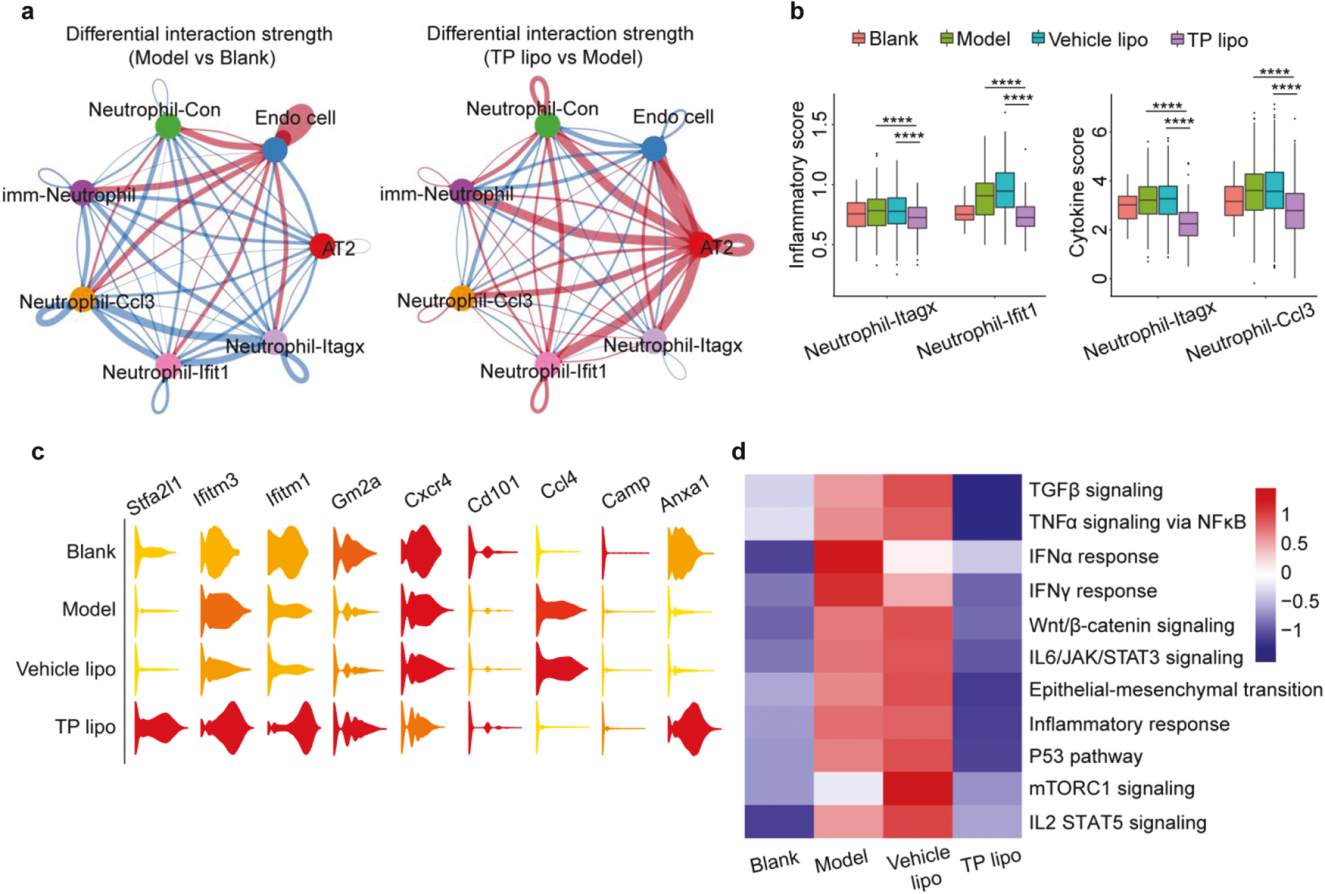
TP lipo reduces the proinflammatory function of pulmonary neutrophils**. a** Differential interaction strength of significant ligand-receptor pairs between any pair of two cell populations. The edge width was proportional to the indicated strength of ligand-receptor pairs. Blue edge weakened cellular communication; Rea edge, strengthened cellular communication. **b** The cytokine score and the inflammatory score of Neutrophil-Itagx and Neutrophil-Ifit1 subpopulations in different groups. **c** Violin plots showing the expression of selected marker genes in four groups. Red, high expression; yellow, low expression. **d** Heatmap showing the expression of functional pathways of neutrophils in different groups. Significance is indicated by ****P ≤ 0.0001

The neutrophils are the first line of defense against infection in the host’s innate immune system. Targeting neutrophils holds promise to lessen the burden of severe COVID-19. Given that lung parenchyma cells such as Endo cells and AT2 cells are the primary targets for early SARS-CoV-2 attack, we used CellChat to investigate the differential interaction strength between neutrophil subsets and lung parenchyma cells. After FA-S infection, Endo cells had strong communication with each neutrophil subset, while there was little communication between neutrophils and AT2 cells (Fig. 7a, left). Conversely, the communication strength of neutrophils-Endo cells was weakened by TP lipo treatment and enhanced between neutrophils-AT2 cells (Fig. 7a, right).

TP lipo was tested for its antiinflammatory and antiviral properties by assessing expression levels of the indicated genes (Fig. 7c). TP lipo has the potential to promote neutrophil apoptosis and inflammation resolution by upregulating the antiinflammatory protein annexin A1 (encoded by the *Anxa1 gene*).^49,50^ However, TP lipo treatment had a minimal effect on the gene encoding tertiary neutrophil granule protein CAP-18 (*Camp*).^40^ The proinflammatory cytokine gene *Ccl4* in neutrophil populations was downregulated significantly by TP lipo, and its expression level was similar to that in the uninfected group. It has been reported that immature CD10^low^CD101^-^CXCR4^+^ neutrophils are amplified in severe COVID-19.^51^ Here, TP lipo treatment increased *CD101* expression and decreased *CXCR4* expression, suggesting that the remission of inflammation and neutrophils may shift from an immature status to a mature status. The elevated expression levels of neutrophil maturation genes (*Gm2a*, *Stfa2l1*) in the TP lipo group confirmed that neutrophils mature during inflammation relief.^40^ *Ifitm1* and *Ifitm3*, encoding interferon-inducible transmembrane (IFITM) proteins, are known for their antiviral effects, such as blocking SARS-CoV-2 entry.^52–54^ We noticed that TP lipo could upregulate the expression of *Ifitm1* and *Ifitm3* on neutrophils, suggesting that TP lipo may enhance the innate antiviral immunity of neutrophils.

Specifically, clusters of Neutrophil-Itagx, Neutrophil-Ifit1 and Neutrophil-Ccl3 are the main sources of the inflammatory cytokine storm (Fig. 7b). The inflammatory levels could be downregulated significantly by TP lipo intervention. We used functional enrichment analysis to understand the molecular pathways involved in inflammation relief. TP lipo downregulated multiple inflammation-related pathways in neutrophils, especially among clusters of Neutrophil-Itagx, Neutrophil-Ifit1, and Neutrophil-Ccl3, including TNFα/NF-κB signaling, IL-6/STAT3 signaling, IFN response, and IL-2/STAT5 signaling (Fig. 7d and S4b–d). These data again showed that TP lipo is a multitarget drug.

## Discussion

This study made several observations regarding severe COVID-19, TP lipo treatment, anti-inflammatory effects, antiviral effects, and pulmonary immunoregulation mechanisms. TP-based therapy has been reported in lung injury and pulmonary fibrosis but is little known for SARS-CoV-2-induced ARDS.^55–57^ Our results reveal the anti-inflammatory and antiviral functions of TP lipo against SARS-CoV-2-induced ARDS in different mouse models. Findings supporting this conclusion are as follows. First, TP was encapsulated in liposomes against its low aqueous solubility and potential toxicity under physiological conditions. Then, two murine ARDS models induced by the Delta variant or WT SARS-CoV-2 were chosen to assay the therapeutic functions of TP lipo. Pulmonary pathological damage caused by each SARS-CoV-2 inoculation was significantly ameliorated by TP lipo, such as lung consolidation, interstitial edema, hyaline membrane formation, cell death, and inflammatory cell infiltration. By 3 dpi, total protein and LDH were elevated in the BALF of the infected mice, along with cytokine storms (IL-3, IL-2, IL-6, IL-10, and G-CSF). The markers that responded to severe COVID-19 were effectively dampened by TP lipo. The LPS-induced ARDS model further confirmed the anti-inflammatory effects of TP lipo in wild-type C57BL6/J mice. Thus, these findings support that TP lipo has powerful antiinflammatory properties against hyperinflammation in severe COVID-19.

We next proved the antiviral effect of TP lipo in animal models and cell models of Delta variant infection. N protein is highly expressed in SARS-CoV-2-infected cells and has been a critical biomarker for COVID-19 diagnosis owing to its high relative abundance and detection sensitivity compared to spike protein.^58,59^ IHC was used to measure the effects of TP lipo on SARS-CoV-2 replication in vivo. The results showed that fewer N protein-positive cells were found in Delta variant-infected mouse lungs with TP lipo treatment. The gRNA and sgRNA (indicative of virus replication) loads were much lower and undetectable in TP lipo-treated mice than in untreated mice. Subsequently, the effects of TP lipo against the Delta variant in vitro were assayed by CPE assay and qRT-PCR (Fig. 2a–d). The cytopathic percentage was dropped dramatically by pretreatment of Vero E6 cells with TP lipo. Thus, both in vivo and in vitro studies have demonstrated that TP lipo exhibits a powerful antiviral effect by inhibiting the replication of live viruses.

There is a reported uncontrolled inflammatory response to COVID-19, as well as NF-B and p38 MAPK activation triggered by the virus.^27,60^ Our results showed that TP lipo suppressed the phosphorylation of NF-κB and p38 MAPK in SARS-CoV-2-infected cells (Fig. 2e), indicating that TP lipo could ameliorate hyperinflammation by targeting upregulated proinflammatory pathways. STAT3 is an antiapoptotic transcription factor and contributes to virus production and pathogenesis by responding to COVID-19-related cytokine storms, such as IL-6, TNF-α, IL-10, G-CSF, and MCP-1.^28,61,62^ STAT3 (Tyr-705) is constitutively phosphorylated in Vero E6 cells during early SARS-CoV and SARS-CoV-2 infections; however, high virus replication after viral infection leads to dephosphorylation of STAT3 (Tyr-705).^28,62^ The Delta variant was reported to have a higher replication efficiency and result in a high virus load and severe disease.^63^ We observed a drastic decrease in p-STAT3 (Tyr-705) in Vero E6 cells 24 h postinfection with the Delta variant, which may be related to cell apoptosis resulting from high virus production.^28,62^ There was an elevated p-STAT3 (Tyr-705) protein level in TP lipo-pretreated Vero E6 cells, suggesting that TP lipo plays a role in inhibiting the continuous replication of the virus by affecting the phosphorylation of STAT3 (Fig. 2e). Therefore, we inferred that TP lipo is a multitarget-directed drug for COVID-19 treatment that regulates the NF-κB, p38 MAPK, and STAT3 pathways.

Single-cell transcriptome profiling was used to reveal how TP lipo modulates FA-S-induced ARDS and pulmonary immune microenvironment heterogeneity. Upon FA-S infection, two activated cell populations (macrophages and neutrophils) were booming for the inflammatory response (Fig. 4d). In detail, Macro-Saa3 cells and Neutrophil-Ccl3 cells played a critical role in hyperinflammation. The two inflammatory immune cell types were highly enriched in inflammation-related genes and were the main sources of cytokine storms. Additionally, these two cell subsets had strong cell-cell communication with pulmonary Endo cells under FA-S infection, which might be related to the endothelial damage caused by the activation of cytokines and chemokines and the extensive recruitment of immune cells.^64^

TP lipo reduced the recruitment of Macro-Saa3 cells and Neutrophil-Ccl3 cells to the lung and downregulated the expression of inflammatory marker genes. In addition, TP lipo increased the interaction strength between AM2 cells and macrophages and neutrophils, suggesting that TP lipo may help to regenerate pulmonary alveoli in COVID-19 patients. The enrichment of inflammation-associated pathways on macrophages and neutrophils was also downregulated in TP lipo-treated ARDS mice. Notably, after acute COVID-19 infection, some COVID-19 survivors experienced physical and neuropsychiatric symptoms for more than 12 weeks.^65^ TP lipo may prevent long COVID-19 syndrome by targeting ICAM-1, IL-6, and IL-2, which are related to vascular injury, inflammation, and chemotaxis (Figs. 6f, 7d, and S3c).^36,48^ Thus, our results uncovered a novel proinflammatory mechanism of SARS-CoV-2 infection and the beneficial antiinflammatory effect of TP lipo treatment for severe COVID-19 infections.

In summary, our molecular, cellular, and animal results revealed that TP lipo could significantly suppress SARS-CoV-2 replication and ameliorate severe ARDS. The scRNA-seq analysis of TP lipo treatment in the SARS-CoV-2-induced ARDS mouse model further broadens our knowledge of immune microenvironment changes in COVID-19 and the anti-inflammatory immunoregulatory ability of TP lipo. TP lipo may hold the potential to become a multitarget drug in the treatment of severe COVID-19.

## Materials and methods

### Animals

During animal studies, Sichuan University’s Animal Care and Use Committee reviewed and approved all procedures (Chengdu, Sichuan, China). Live SARS-CoV-2 virus was used in all procedures that required approval from the Institutional Animal Care and Use Committee of the Institute of Medical Biology, Chinese Academy of Medical Science. These procedures involving live SARS-CoV-2 virus were carried out in the ABSL-4 facility of Kunming National High-level Biosafety Primate Research Center.

### Preparation and characterization of liposomes

TP lipo was composed of 1,2-distearoyl-sn-glycero-3-phosphocholine (DSPC)/cholesterol/distearoylphosphatidyletanolaminemethyl-PEG 2000 (DSPEmPEG2000) at a mole ratio of 6:3:1 (DSPC was from Avanti, USA; cholesterol was from Sigma-Aldrich, USA; DSPEmPEG2000 originated from AVT, China). The drug-to-lipid ratio of tripterin to total lipid was 1 mg/40 mg. TP lipo and Vehicle lipo were prepared by the lipid film hydration and extrusion methods modified from protocols published previously.^19,66^ Briefly, select lipids with or without tripterin were placed in a round-bottom flask and dissolved in chloroform/methanol (2:1 v/v) organic solvent. The lipid film was obtained from a Rotavapor R-300 system (BUCHI, Switzerland) (55 °C, 50 rpm, 250 kPa, 1 h). Subsequently, normal saline (1 mL/20 mg total lipids) was added to hydrate the dried lipids with a vigorous vortex (55 °C, 1 h). An extruder (610000, Avanti, USA) was used to extrude suspensions through three different sizes of polycarbonate membranes (400, 200, and 100 nm) (610007, 61006, 61005, Avanti, USA) to obtain unilamellar liposomes with good homogeneity. The determinations of the size distribution, and PDI was calculated using dynamic light scattering (DLS) (Malvern Zetasizer Nano ZS, UK). Transmission electron microscopy was used to sample liposome surface morphologies (TEM) HT7800 (HITACHI, Japan).

### Preparation of FA-S

The preparation procedure of FA-S was described previously.^29^ Briefly, the SARS-CoV-2 isolate GD 108# strain obtained from Guangdong Provincial Center for Disease Control and Prevention was propagated in African green monkey kidney cells (Vero E6). The monolayer of cultured Vero E6 cells was maintained in Dulbecco’s modified Eagle’s medium (DMEM) from Gibco, USA. The formaldehyde fixation of the SARS-CoV-2 virus was performed at 4 °C in the ABSL-4 facility. Next, to remove the formaldehyde, the virus was ultrafiltered by a regenerated cellulose membrane with 3 kDa NMWCO (Millipore, USA). The FA-S was resuspended in PBS and stored at 4 °C until further use.

### Mice and experimental procedures

Male C57BL/6 mice (8–10 weeks) were purchased from Beijing Vital River Laboratory Animal Technology Company. Transgenic hACE2 mice (8–10 weeks) on a C57BL/6 background were obtained from the National Institutes for Food and Drug Control. All mice were housed in a specific-pathogen-free (SPF) facility. To establish the Delta variant-induced ARDS model, hACE2 mice were anesthetized with 5% isoflurane and then intratracheally instilled with 2 × 10^5^ PFU of Delta variant live virus in 50 μl of PBS per mouse using a 29-gauge insulin syringe. To establish the FA-S-induced ARDS model, hACE2 mice were anesthetized with 5% isoflurane and then intratracheally instilled with 2 × 10^6^ PFU of FA-S in 50 μL of PBS per mouse using a 29-gauge insulin syringe. To establish the LPS-induced ARDS model, WT mice were anesthetized with 5% isoflurane and then intratracheally instilled with 5 mg/kg LPS in 50 μL of PBS per mouse using a 29-gauge insulin syringe.

To assess the therapeutic effects of TP lipo in the Delta variant-induced ARDS model, 150 μL of TP lipo (1.5 mg/kg), Vehicle lipo, or 0.9% NaCl was intraperitoneally injected into hACE2 mice 0.5, 24, 48 h after intratracheal instillation of Delta variant live virus. Mice were euthanized 96 h post-instillation for lung processing.

To assess the therapeutic effects of TP lipo in the FA-S-induced ARDS model, 150 μL of different doses of TP lipo (0.1 mg/kg or 1.5 mg/kg), Vehicle lipo, or 0.9% NaCl was intraperitoneally injected into hACE2 mice 0.5, 24, 48 h after intratracheal instillation of FA-S. Mice were euthanized 72 h post-instillation for lung processing or collection of BALF.

To assess the therapeutic effects of TP lipo in the LPS-induced ARDS model, 150 μL of different doses of TP lipo (0.1 mg/kg or 1.5 mg/kg), Vehicle lipo, or 0.9% NaCl was intraperitoneally injected into hACE2 mice 0.5, 24, 48 h after intratracheal instillation of LPS. Mice were euthanized 72 h post-instillation for lung processing or collection of BALF.

### BALF harvesting and analysis

Mice’s BALF was collected by cannulating their trachea and leaving their lungs with 1 mL of cold PBS. The immune cells in BALF were stained with a Diff-Quick Stain Kit (Solarbio, China). Briefly, smears of BALF were fixed with Diff-Quik fixative for 20 s. Next, after staining the slides with Diff-Quik I for 20 s and Diff-Quik II for 3 s, they were rinsed with distilled water and inspected under a microscope. According to the manufacturer’s instructions, total protein levels were determined using a PierceTM BCA Protein Assay Kit (Thermo Scientific, USA). The LDH levels were determined using the CytoTox 96 nonradioactive cytotoxicity assay kit from Promega, USA. The procedures were all performed in the dark.

### Cell viability assay

To investigate the cytotoxicity of TP lipo. Vero E6 cells were tested for viability by the CCK-8 assay after 48 h of coincubation with Vehicle lipo or TP lipo at the indicated concentrations. Untreated cells or Vero E6 cells coincubated with 10% DMSO were used as control groups. Briefly, the cells (5 × 10^4^ ml^−1^, 100 μL) were seeded on 96-well plates, incubated for 12 h and then treated for 48 h with varying concentrations of drugs. Next, an enzyme-linked immunoassay analyzer was used to measure the absorbance at 450 nm after adding CCK-8 reagent (10 μL/well) for another 1 h (BioTek Synergy 4, USA).

### Neutralization of Delta variant live virus

To assess the neutralization of Delta variant live virus infection, Vero E6 cells (5 × 10^4^) were preincubated with Vehicle lipo or TP lipo at the indicated concentrations (0, 125, 250, 500 nM) for 1 h at 37 °C and then exposed to the Delta variant (MOI = 0.05). Nontreated cells and cells incubated with 500 nM Vehicle lipo or 500 nM TP lipo without viral exposure were used as control groups. The CPE were recorded under a microscope 24, 48, and 72 h after viral exposure. The percentage of CPE at the indicated time points was analyzed from three independent experiments. The mRNA levels of Delta variant gRNA and sgRNA in Vero E6 cells and the cell culture supernatant were analyzed by qRT-PCR 24 and 72 h after viral exposure, respectively. The primer and probe sequences used for gRNA (forwards, 5′- GACCCCAAAATCAGCGAAAT -3′;

reverse, 5′-TCTGGTTACTGCCAGTTGAATCTG-3′;

probe, 5′-FAM-ACCCCGCATTACGTTTGGTGGACC-MGB-3′), and sgRNA (forwards, 5′-CGATCTCTTGTAGATCTGTTCTC-3′;

reverse, 5′-ATATTGCAGCAGTACGCACACA-3′;

probe, 5′-FAM-CGAAGCGCAGTAAGGATGGCTAGTG-MGB-3′) were selected according to the sequences recommended by the WHO and China CDC.

### Western blot analysis

To extract the proteins from Vero E6 cells, the supernatant of the Vero E6 cell culture was discarded, and the cells were lysed and denatured by adding sample buffer and boiling for 10 min. To extract the proteins from lung tissue, 50 mg of lung tissue from each mouse was added to 500 μL of 1 mM RIPA lysis buffer (Beyotime, China, P0013B) supplemented with 10 μL protease Inhibitor cocktail (MedChemExpress, HY-K0010) and 10 μL Phosphatase Inhibitor Cocktail II (MedChemExpress, HY-K0022) and then ground by High-Speed Tissue Homogenizer (Servicebio, KZ-II). The lysates were collected and centrifuged at 13,000 rpm for 10 min. Then, each sample containing 2 mg of protein was mixed with 125 μL of SDS-PAGE Sample Loading Buffer (Beyotime, P0015 L) and boiled for 10 min.

Proteins were loaded (10 μL/lane), resolved by 8% sodium dodecyl sulfate (SDS)-polyacrylamide gel electrophoresis (PAGE), and then transferred to PVDF membranes (Millipore). After blocking with 5% nonfat milk in Tris-buffered saline containing 0.05% Tween 20 (TBST) for 1 h at room temperature. Next, the membranes were probed overnight at 4 °C with the following primary antibodies: mouse polyclonal anti-p-p38 MAPK (T180/Y182) (Cell Signaling Technology, 1:2000), rabbit polyclonal anti-GAPDH (Santa Cruz Biotechnology, 1:4000), rabbit polyclonal anti-STAT3 (HUABIO, 1:1000), rabbit polyclonal anti-p-STAT3 (Tyr705) (HUABIO, 1:500), rabbit polyclonal anti-p-NF-κB p65 (S536) (Cell Signaling Technology, 1:1000), rabbit polyclonal anti-p38 MAPK (Cell Signaling Technology, 1:2500), rabbit polyclonal anti-NF-κB p65 (Cell Signaling Technology, 1:1000), and mouse polyclonal anti-Vinculin (Sigma, 1:4000). After washing with TBST three times, the membranes were incubated with horseradish peroxidase (HRP)-conjugated anti-rabbit secondary antibodies or anti-mouse antibodies for 1 h at room temperature (Cell Signaling Technology, 1:5,000). The membranes were then developed using an enhanced chemiluminescence detection kit (Millipore) and exposed to autoradiography film with a film developer in a dark room. Vinculin was used as an internal control.

### Cytokine screening and analysis

At the indicated times, the mice’s BALF was collected. BALF samples (50 µL) were screened with a Luminex Mouse Cytokine 23-plex (IL-3, IL-2, IL-13, IL-6, IL-4, G-CSF, RANTES, IL-10, Eotaxin, IL-12p40, IL-12p70, and so on).

### Preparation of lung single-cell suspensions

Four groups of hACE2 mice were used for scRNA-seq, including the ARDS model induced by intratracheal instillation of FA-S and the blank group undergoing PBS, as well as the ARDS model treated with vehicle lipo or TP lipo. Three mice were used for each group. 4% chloral hydrate was used to euthanize mice. Then, perfusing the heart until the lungs became pale reduced the circulation of contaminated blood cells. The lung tissue was dissected from mouse and minced into smaller pieces below 1 mm^3^ on ice. A lung dissociation kit was applied to generate single-cell suspensions from mouse lungs (Miltenyi Biotec, order no. 130-095-927). Different amounts of DNA enzymes were used according to the tissue homogenate viscosity. After digestion, 70 μm and 40 μm nylon mesh filters (Corning) were used to filter out undigested tissue clumps. Other red blood cells (RBCs) were cleared by RBC lysate (Miltenyi). Cell viability and counting were monitored with AO/PI double staining reagent by microscopy. Cell debris and necrotic cells were removed with a Cell Debris Removal Solution and Dead Cell Removal Kit (Miltenyi). We used samples with viability greater than 90% for further sequencing. In less than 2 hours, the cells were ready for loading onto the 10× Chromium controller.

### Single-cell RNA library construction and sequencing

Cells were resuspended in 1×PBS containing 0.05% BSA at a concentration of 1 × 10^6^/mL for scRNA-seq. Cell suspensions were captured by the 10× Genomics platform (Pleasanton, CA, USA). RNA libraries were constructed using the Single Cell 3′ Library Kit V3.1 (Chromium Next GEM). A 10× Genomics-based droplet sequencing approach was used to detect transcriptome profiles in individual cells. Using a NovaSeq 6000 Sequencer (Illumina) at the high output with 150-base paired dual-end sequencing reads, libraries were prepared and sequenced.

### scRNA-seq data processing

The original sequencing data were trimmed by Fastp to remove adapter sequences and low-quality reads, and basic statistical data were collected. Data from the sequencing were aligned and clustered, and gene expression was analyzed using the single-cell sequencing software Cell Ranger (version 6.1.2). The Seurat R software package (version 4.0.1) was used to filter out cells with a total number of genes greater than 6000, cells with a gene number of less than 200, cells with a proportion of mitochondrial genes greater than 10%, and cells with a balance of hemoglobin genes greater than 1%. Data normalization and identification of variable genes were realized by the CellCycleScoring algorithm and the FindVariableGenes algorithm. On the graphs for the diffusion structure scoring of each cell, the Louvain algorithm was applied to cluster the cells. Furthermore, the results of cell clustering were visualized using the UMAP. We used Seurat bimod and SingleR to annotate cell types. By analyzing transcriptional changes in cells, Monocle2 performed a pseudotime analysis to determine differentiation trajectories. To identify enriched data sets, Gene Ontology (GO), Kyoto Encyclopedia of Genes and Genomes (KEGG) enrichment, and HALLMARK data sets were examined. CellChat packages were used to determine the potential interactions between different immune cells.

### Histology and immunofluorescence

The lungs were fixed in 4% paraformaldehyde at room temperature for two days, embedded in paraffin, and sectioned at 3 µm. The severity of the lung damage was evaluated by the use of H&E staining in accordance with a previously described scoring system from least severe to most severe.^22^ Assays were conducted with DeadEndTM Fluorometric TUNEL System (Promega, USA) to identify apoptotic cells in lung tissues. The antigens were extracted from paraffin-embedded sections by incubating them in EDTA buffer after blocking endogenous peroxidases with 3% H_2_O_2_. Blocking buffer (5% goat serum) was then applied to the sections. Primary antibodies used for immunohistochemistry (IHC) or immunofluorescence analysis included rabbit anti-lymphocyte antigen 6 complex locus G6D (anti-Ly6G, BD Biosciences, 551459) and rabbit anti-N protein (Sino Biological, 40143-R001).

### Statistical analysis

All data were analyzed using one-way ANOVA or Student’s unpaired *t*-test (GraphPad InStat Software Inc., CA, USA). The results are presented as the means ± SEM. *P* < 0.05 is considered significant (significance is denoted as follows: ns no significance; ^*^P ≤ 0.05; ^**^P ≤ 0.01; ^***^P ≤ 0.001; ^****^P ≤ 0.0001).

## Supplementary information

revised supplementary materials-R2

Data S1. Marker genes of myeloid cells and neutrophils

## Data Availability

The authors declare that there are no primary data sets or computer codes associated with this study. All data and materials are available to the researchers once published.
