# Supplementary material for: Tripterin liposome relieves severe acute respiratory syndrome as a potent COVID-19 treatment
Source: Signal Transduct Target Ther. 2022 Dec 24;7:399. doi: 10.1038/s41392-022-01283-6 (PMC9789731; doi:10.1038/s41392-022-01283-6)
Supplement: Supplementary file 1 — revised supplementary materials-R2 [file 41392_2022_1283_MOESM1_ESM.docx]

Supplementary Materials for

Tripterin liposome relieves severe acute respiratory syndrome as a potent COVID-19 treatment

Haiying Que^1#^, Weiqi Hong^1#^, Tianxia Lan^1#^, Hao Zeng^1#^, Li Chen^1^, Dandan Wan^1^, Zhenfei Bi^1^, Wenyan Ren^1^, Min Luo^1^, Jingyun Yang^1^, Cai He^1^, Ailing Zhong^1^, Xiawei Wei^1*^

Correspondence to: xiaweiwei@scu.edu.cn

**This PDF file includes:**

Figures. S1 to S4

**Other Supplementary Materials for this manuscript include the following:**

Data S1: Marker genes of myeloid cells and neutrophils

Figure S1.


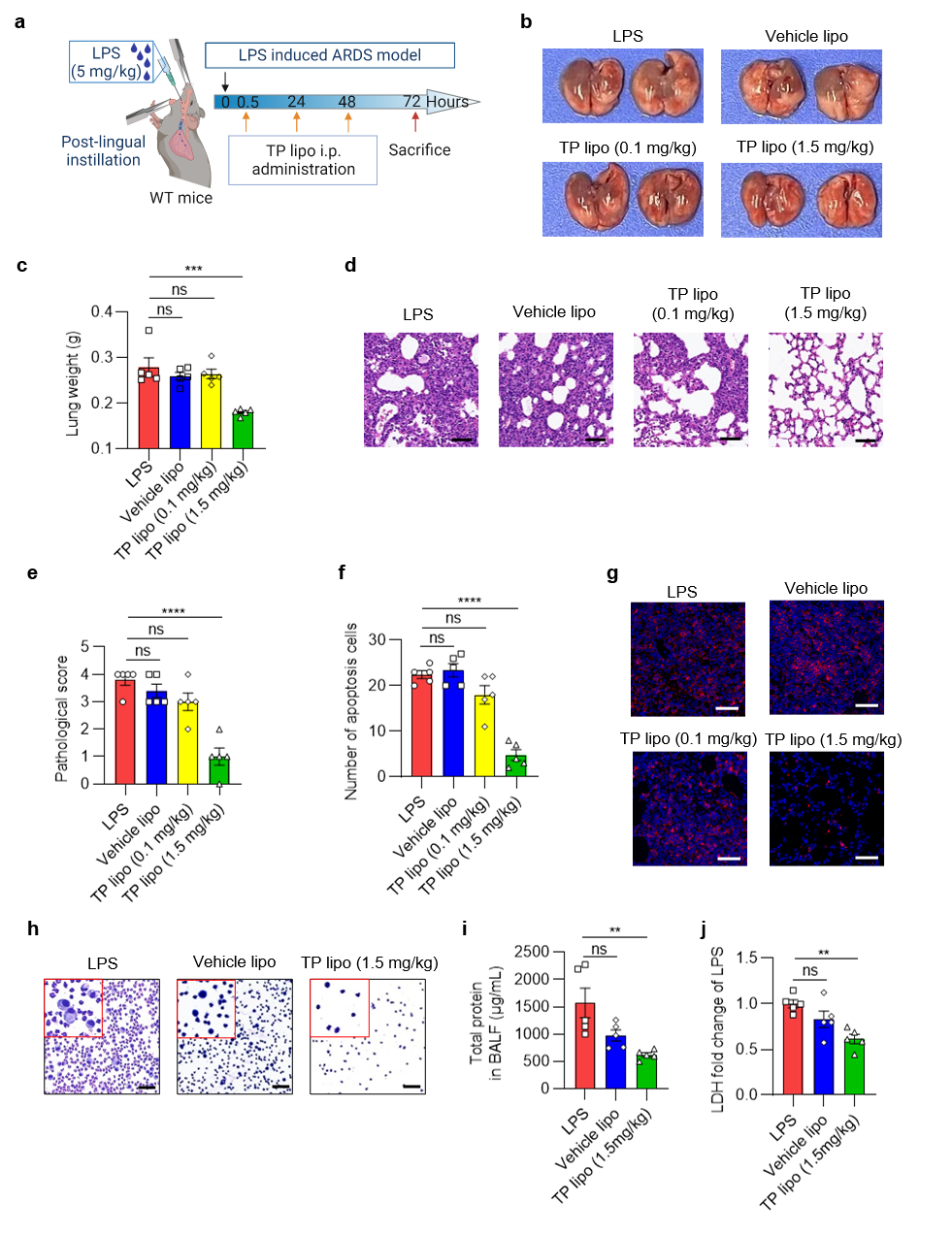


**Figure. S1** TP lipo ameliorates ARDS induced by LPS in WT C57BL/6 mice. **a** Protocol of the WT mouse ARDS model induced by LPS. **b**, **c** Images of lung tissues (**b**) and changes in lung weight (**c**). **d, e** H&E staining (**d**, scale bar represents 50 μm) and pathological score (**e**) in representative mouse lung sections. **f** Number of apoptosis cells obtained from TUNEL staining. **g** Typical images of immunofluorescence analysis for Ly6G positive neutrophils. The scale bar represents 50 μm. **h** Typical images of cells in BALF obtained from Diff-Quik staining. The scale bar represents 50 μm**. i**, **j** Total protein levels (**i**) and fold change of LDH (**j**) in BALF. Data represent the mean ± SEM; n=5. Significance is indicated by: ns, no significance; ***P* ≤ 0.01; ****P* ≤ 0.005; *****P* ≤ 0.0001.

Figure S2.


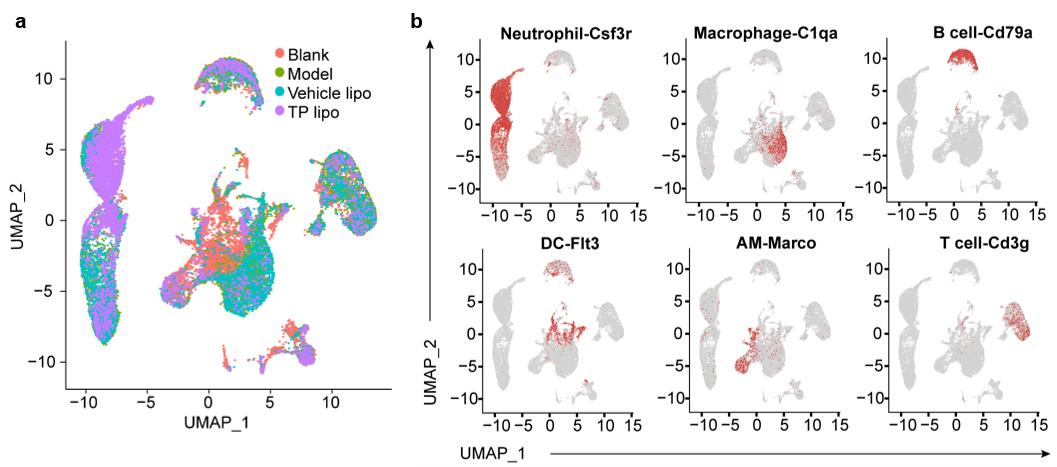


**Figure. S2** Overview of all clusters in the lung. **a** UMAP plot showed the group distribution of single cells analyzed by 10 × scRNA seq. **b** Expression patterns of the marker genes of Neutrophil (Csf3r), Macrophage (C1qa), B cell (Cd79a), DC (Flt3), AM (Macro), and T cell (Cd3g) projected on UMAP plot. Red, high expression; Gray, low expression.

Figure S3.


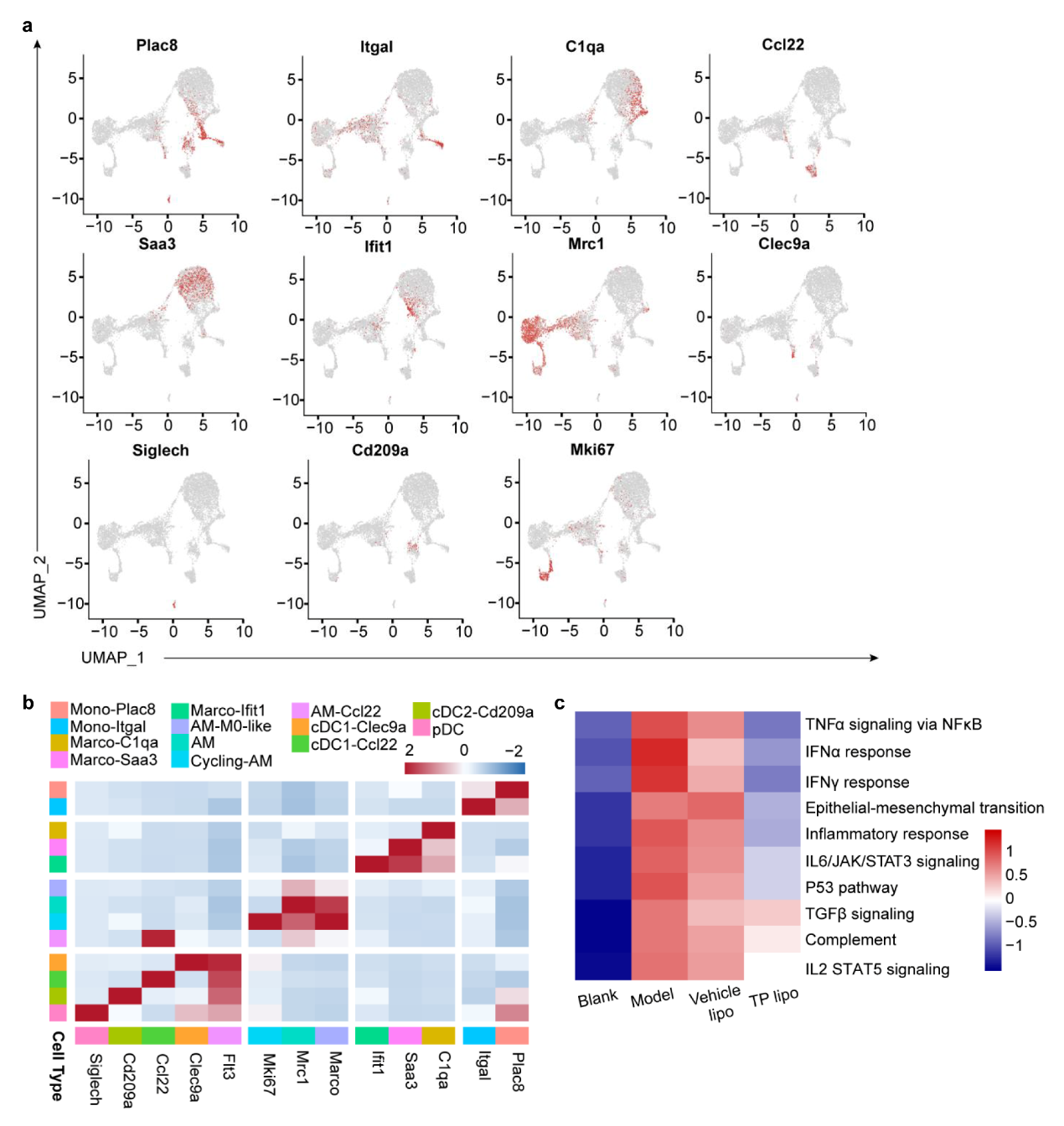


**Figure. S3** DEGs in each cluster among myeloid cells and functional pathway analysis in a different group. **a** UMAP plot of the marker gene in the indicated myeloid cell cluster. Red, high expression; Gray, low expression. **b** Heatmap of the top transcripts in each cluster showed clear demarcation between different clusters. **c** Heat map showing the expression of functional pathways of myeloid cells in each group.

Figure S4.


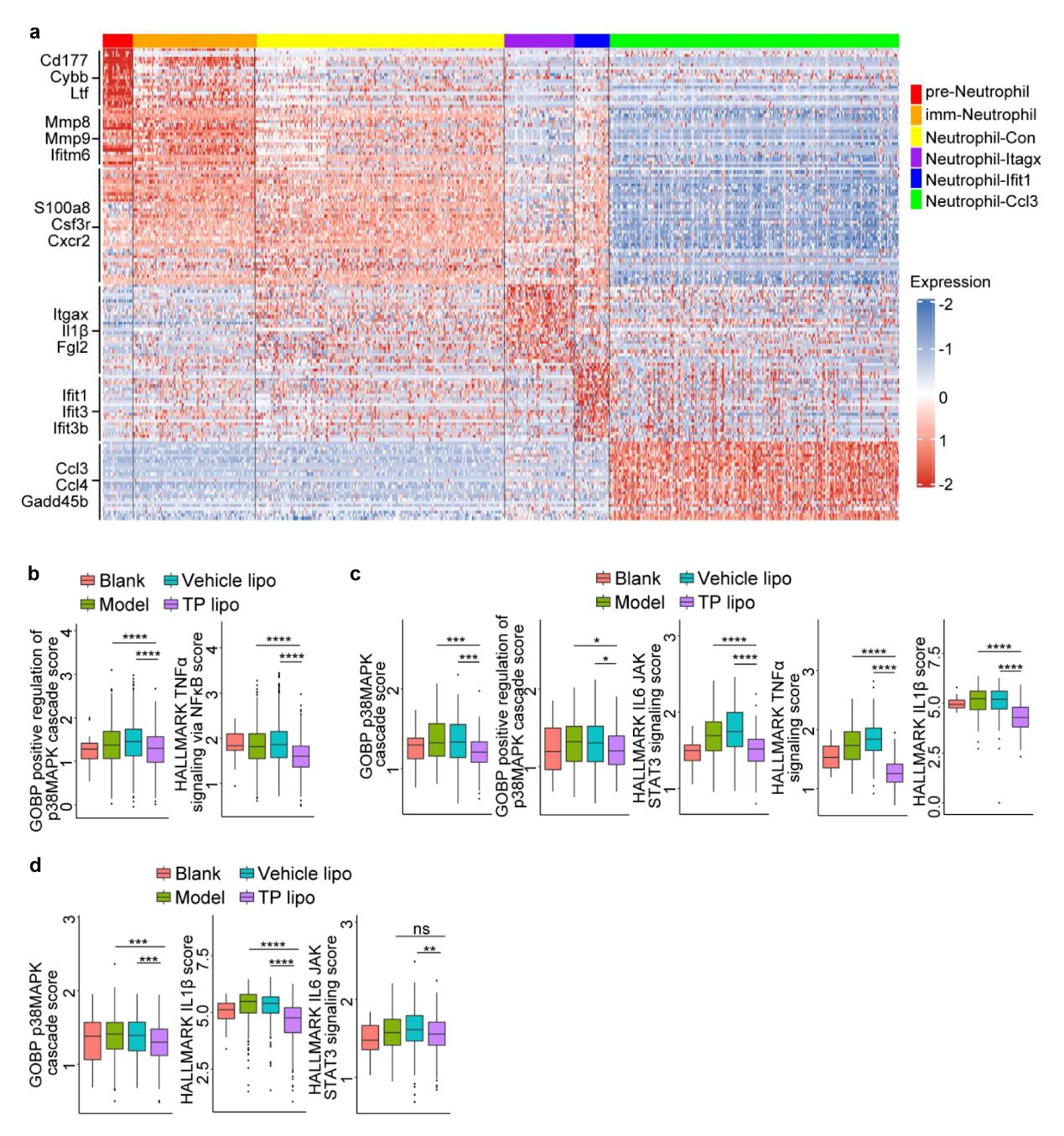


**Figure. S4** DEGs in each cluster among neutrophil and representative pathway scores in different groups. **a** Heat map of marker genes enriched in each neutrophil cluster. Red, high expression; Blue, low expression. **b-d** Inflammatory-related pathway scores of Neutrophil-Ccl3 (**b**), Neutrophil-Ifit1 (**c**), and Neutrophil-Itgax (**d**) among Blank controls, Model, Vehicle lipo and TP lipo groups. Significance is indicated by: ns, no significance; **P* ≤ 0.05; ***P* ≤ 0.01; ****P* ≤ 0.005; *****P* ≤ 0.0001.
